# Uncovering the Molecular Signatures of Rare Genetic Diseases in the Punjabi Population

**DOI:** 10.3390/ijms27010206

**Published:** 2025-12-24

**Authors:** Iqra Tabassum, Muhammad Shafique, Muhammad Shoaib Akhtar

**Affiliations:** 1 Forensic DNA Typing Laboratory, National Center of Excellence in Molecular Biology, University of Punjab, Lahore 53700, Pakistan; iqra.tabassum@cemb.edu.pk (I.T.); shafique@cemb.edu.pk (M.S.); 2 Punjab Thalassemia and Other Genetic Disorders Prevention and Research Institute, Sir Ganga Ram Hospital, Lahore 54000, Pakistan; 3 Department of Cell Biology and Human Anatomy, School of Medicine, University of California Davis, Davis, CA 95616, USA; 4 Department of Integrated Biosciences, Graduate School of Frontier Sciences, The University of Tokyo, 5-1-5 Kashiwanoha, Kashiwa 277-8562, Chiba, Japan

**Keywords:** consanguinity, pathogenic variants, Punjabi population, rare genetic diseases, sequencing

## Abstract

Rare genetic diseases (RGDs) affect individuals, families, and healthcare systems worldwide. Population-scale genomic data remain largely restricted to Western cohorts with an estimated 10,000 RGDs. South Asian populations remain underrepresented in molecular, clinical, and genomic databases. This study presents the first preliminary molecular genetic characterization of RGDs in the Punjabi population of Pakistan. Data were collected from the provincial RGD registry at the Punjab Thalassemia and Other Genetic Disorders Prevention and Research Institute (PTGDPRI), Lahore. Families diagnosed using next-generation sequencing (NGS) between 2021 and 2023 were enrolled. Structured questionnaires captured clinical, demographic, and socioeconomic information, and statistical and genetic analyses were performed to assess allele frequencies, and disease distribution. The registry included 167 families with 72 distinct RGDs, with a mean burden of 0.81 ± 0.24 affected children per family. Niemann–Pick disease (NP), progressive familial intrahepatic cholestasis (PFIC), and mucopolysaccharidosis (MPS) were the most common diseases. Consanguinity was observed in 89% of families, 77% of which involved first-cousin marriages, and was significantly associated with RGD incidence. Most families belonged to low-income groups despite high literacy rates, underscoring inequity in healthcare. The primary and secondary variants included 131 variants, including copy number variants (CNVs) and single nucleotide variants (SNVs), annotated as pathogenic, likely pathogenic, or variants of unknown significance (VUS) across 109 genes, including 24 South Asian-enriched variants. This study provides the first genomic and epidemiological overview of RGDs in the Punjabi population. The findings reveal how genetic, socioeconomic, and cultural factors converge to amplify the RGD burden and highlight the need for affordable molecular diagnostics, inclusive genomic databases, and regional genomic surveillance initiatives in South Asia.

## 1. Introduction

An RGD occurs intermittently or rarely in the general population. Rarity means only a handful of patients are affected by the disease. The number of estimated RGDs is ~10,000 [1], which are caused by both genetic and somatic variations. Characterization of RGDs is challenging due to the rarity of incidence, and they remain mysterious. However, advancing technologies and collaborative efforts of experts are providing novel information to resolve this mystery. In Orphanet database, 6172 unique RGDs are present [2]. Of these 6172, 3510 are of pediatric onset, 600 of adult onset, and 908 of onset spanning both pediatric and adult groups. Most RGDs occur as natural genetic defects or from missing heritability [2,3] and are genetically classified as monogenic, polygenic, oligogenic, or chromosomal anomalies [4]. RGDs affect approximately 10% of the population [5]. In some studies, the survey point prevalence was expected to be approximately 6.53%, 0.34%, and 0.30% in random populations for common, rare, and ultra-rare genetic diseases, respectively. RGDs can be grouped into metabolic, neurological, or developmental diseases based on their pathology and phenotype [6]. Diseases that affect fewer than 20 people globally are called ultra-rare diseases [7].

The definition of RGDs varies across different geographic regions and healthcare systems [8], but an international definition provided by the World Health Organization (WHO) states that RGD is a medical condition with a specific pattern of signs, symptoms, and clinical findings affecting less than or equal to 1 in 2000 (50/100,000) people in any region [9].

From a medical perspective, the characterization of an RGD depends on the broader diversity of disease and symptoms that can vary from disease to disease as well as within the same disease [10]. The same disease can have variations in clinical manifestations from person to person, which are differentiated into subtypes of the same disease, and it remains challenging in the diagnostic journey due to variable factors [11]. Furthermore, each RGD has a different effect on life expectancy: some are fatal at birth, some are degenerative and life-threatening, whereas others are compatible with a normal life if diagnosed in time and properly managed and/or treated [12].

Due to the wide range of diversity and complexity, it is difficult to properly recognize an RGD at earlier stages, resulting in a longer diagnostic odyssey [13,14,15]. Generally, the first line of diagnosis is a clinical diagnosis, including routine hematological and biochemical tests and radiographic examinations. Once a provisional diagnosis is established, confirmatory diagnostic tests are necessary to make a definitive diagnosis. Classically, biochemistry and histopathology practices have been used to identify pathology at the protein level. Recently, karyotyping and in situ hybridization methods have been applied on a vast scale to identify pathologies at the genetic level. These methods, together with polymerase chain reaction (PCR)-based assays, have been widely accepted for their target-oriented accuracy [16]. However, the etiology of many RGDs has not yet been established. This unknown etiology has led to the use of advanced genomic techniques for RGD diagnosis. Despite the known variants, there are unknown variants that probably have 10–90% of pathogenic effects [17]. The growing chances of ambiguity in the results of RGD are due to these unknown variants called VUS.

Recently, short- and long-read NGS [18,19] and single-cell genomic technologies [20] have been used for RGD diagnosis. NGS has accelerated the precise diagnosis of RGDs with a confirmed outcome of 25–50% [21]. Comparative analysis of clinical and NGS data, together with advanced bioinformatics methods, has proven to be a powerful technique for accurate diagnosis [22]. Three types of NGS approaches have been in practice: whole-genome sequencing (WGS), whole-exome sequencing (WES), and targeted-capture sequencing. These approaches can be used for both short-variant discovery and CNVs [23]. WES is a leading diagnostic strategy [24]; however, one-third of RGDs remain undiagnosed because of technical limitations in determining variations in non-coding regulatory genomic regions [25]. To overcome these limitations of WES, WGS can identify non-coding region variants and is able to identify 99% of the significant risk of RGD [26,27] in addition to those possible with WES [28,29,30,31,32]. However, in cases when a provisional diagnosis is strong and the patient had a family history of a particular disease, it is possible to utilize targeted-capture sequencing instead of WGS and WES. In addition to these conventional NGS applications, single-cell genomic technologies have the potential to identify pathologies at the cellular level. Single-cell technology defines cellular heterogeneity at the cell level; however, specialized computational tools are required for comparison with disease phenotypes [33].

Despite the availability of these advanced technologies, RGD diagnosis remains a challenge because these technologies have not been optimized for this purpose, owing to limited testing options. Challenges in RGD diagnosis also lead to challenges in treatment. Most RGDs involve neurodevelopmental and metabolic pathologies [34]. Affected patients are on a life-death race, and death tolls always remain high. Higher mortality and limited lifespans have made it challenging to devise treatments using conventional methods [35,36]. Most RGDs do not have a treatment, but different therapeutic regimens are used only to manage the clinical presentation [37,38,39,40,41]. Due to the rarity of RGDs, drug development is challenging, and medicinal treatment exists for only 5% of RGDs to date [2]. However, recent advances in genomics and drug development have opened new avenues for discovery. Moreover, biobanking, organ-on-chip devices, and in silico docking technologies have the potential to validate novel drugs [42].

It is worth noting that among the general population, six to eight people out of a hundred are carriers of RGDs. There is no significance of these carrier individuals, but if two individuals marry and carry the same genetic abnormality, there is a 25% chance of inheriting a genetic abnormality per pregnancy. It can be ‘’frequent’’ to be affected by an RGD in a family carrying RGD but it is “rare” to find a family carrying RGD [12]. Marrying a cousin (consanguinity) in such families can lead to a higher incidence of RGD in offspring. The consanguinity rate varies among populations based on religion, culture, and politics. The global distribution of consanguinity is 10.4% of the entire population [43]. Consanguineous marriages are prevalent in North Africa, the West, and South Asia. The consanguinity rates of some countries in this region are reported as Saudi Arabia, 30% [44], Libya, 38% [45], Qatar, 54% [46], and Pakistan over 80% [47,48]. The consanguinity level is comparatively low (1–1.5%) in European, South American, and Australian populations, depending on the social demographic effect [49,50].

Numerous barriers to RGD patients and carriers are observed in developing countries. Pakistan is one such country where RGD patients suffer from socioeconomic crises. In addition, the Pakistani population has a consanguinity rate of more than 80%. This higher consanguinity at the population scale increases the risk of genetic diseases, including RGD [47,51,52,53,54]. A high consanguinity rate leads to reproductive loss, risk of abortion, and neonatal or postnatal deaths [55]. However, no statistics exist on the incidence of RGDs in the Pakistani population. It is important to look for existing RGDs, causal genes, pathogenic variants, and their relationships with demographic factors in the Pakistani population [56]. The Pakistani population generally comprises Caucasian ethnic groups [57]. The major ethnic groups include Punjabi, Pathan, Sindhi, Saraiki, Balochi, Kashmiri, and Kalash. Punjabi people comprise the largest group, with approximately 150 million people. Punjabi people not only live in Pakistan but also have major populations in India (~37 million), Canada (~1 million), United States (~0.25 million), and England (~0.75 million).

All these Punjabi populations were homed in the Punjab state of British India until 1947, when it was divided into two countries, India and Pakistan. Following this partition, many Punjabis moved out of sub-continent due to religious, political, or identity crises. Many of these migrating populations moved to the United States, Canada, England, and other European countries only one or two generations ago and thus share the same genetic background as the Pakistani and Indian Punjabi populations. In Pakistani Punjab, the majority of Punjabi people practice Islam and are practicing Muslims, while in India, the majority of Punjabi follow Sikhism. Sikhism prohibits consanguinity up to seven generations, but it is common in Pakistani Punjab. This is the reason why Pakistani Punjabi have consanguinity rates of over 80% [47], while in Indian Punjab, it is less than 5% [58]. This higher consanguinity is not only common among Pakistani Punjabi population but also in the Muslim world in general [59]. It is not only religion but also culture, family wealth, power politics, and other associated factors that have historically led to increased consanguinity practices.

In this study, we reported a recently built RGD provincial registry in the Punjab region of Pakistan. We registered the maximum possible number of families of Punjabi origin with confirmed RGD diagnosis and obtained information on RGD, causal gene, pathogenic variant, consanguinity, and other demographic factors. Compiling all this data, we presented the incidence of RGDs, causal genes, pathogenic variants, and the role of consanguinity during 2021–2023 in this manuscript.

## 2. Results

### 2.1. Identification and Incidence of RGDs

The PTGDPRI is the only public institute dedicated to RGDs in Punjab and Pakistan. This institute is run by the provincial government of Punjab and has maintained an RGD registry since January 2021. We have presented data spanning 2021–2023 in this study. We registered patients visiting the PTGDPRI for clinical services, including diagnosis, prenatal screening, or genetic counselling. Although this institute is a provincial body, patients from other geographic regions and provinces of the country also visit for clinical services (Supplementary Figure S1A). RGDs are broadly distributed across many districts, but most RGD-city combinations involve only a single affected family. However, certain cities, such as Lahore, Faisalabad and Rawalpindi, which are metropolitan, show clusters of higher frequencies for specific RGDs, with up to six cases observed in some instances. These data underscore the importance of region-wide genetic screening, as RGDs are present throughout the country (Supplementary Figure S1B).

In our data spanning 36 months from January 2021 to December 2023, 167 families visited the PTGDPRI. These families presented 72 RGDs of pediatric onset as the primary diagnosis, with the final diagnosis made within the first four years of life. A list of these 72 RGDs along with their incidence within the current registry is provided in Supplementary Table S1. These 167 families represented 118 probands and 391 children. Of the 391 children, 293 were affected by RGDs and 188 had died. Of these 188 children who died, 70 died without any diagnosis, including both pre- and post-mortem investigations.

All 167 families were aware of the incidence of RGD in the family and underwent CVS for an ongoing pregnancy. In the first year, 76 families were registered, and no genetic data were recorded; instead, a clinical final diagnosis (confirmed on genetic testing) with a referral registration form submitted by the referring clinician’s office was registered. These 76 RGD families were diagnosed with 19 different RGDs, as identified in Supplementary Table S1. However, the registry started registering genetic data in the second year, and 91 families submitted detailed genetic data at the time of registration. All 91 families who submitted genetic data were tested using massive parallel NGS for short and structural variants from a College of American Pathologists (CAP) and/or ISO15189 accredited genetic testing laboratory. Short variant testing was performed for 91 families, and 35 families were tested for structural variants in addition to short variant testing. Among the structural variants, CNVs were detected using sequencing depth. Eight of these families underwent WGS, 56 underwent WES, and 27 underwent targeted-capture sequencing. The mean sequencing depth for WGS was 30X, while it was 20X for WES and targeted-capture sequencing. This information is presented in Table 1. In 91 cases, 72 primary and 34 secondary variants were associated with RGDs annotated under the joint standards and guidelines of the American College of Medical Genetics (ACMG) and the Association of Molecular Pathology (AMP) [17], Sequence Variant Interpretation (SVI) recommendations for ACMG/AMP guidelines from The ClinGen SVI Group, and/or ACMG secondary findings (SF) version 3.0 [60] (Supplementary Table S2A). All these RGDs showed an autosomal recessive inheritance pattern, except for adrenoleukodystrophy, glucose-6-phosphate dehydrogenase (G6PD) deficiency, hemophilia, and intellectual disability type 5, which are known to be X-linked recessive. These RGDs were caused by pathogenic, likely pathogenic or VUS in 109 genes (Supplementary Table S2B,C). A primary variant was identified in all 91 cases, with 58 secondary variants and 31 VUS (Supplementary Table S2D). All these variants were SNVs except one CNV in TJP2 gene. A geneticist at the registry reviewed all variant annotations to ensure consistency within the registry. A summary of the pathogenic classification of these variants is provided in Table 2.

**Figure 1:**
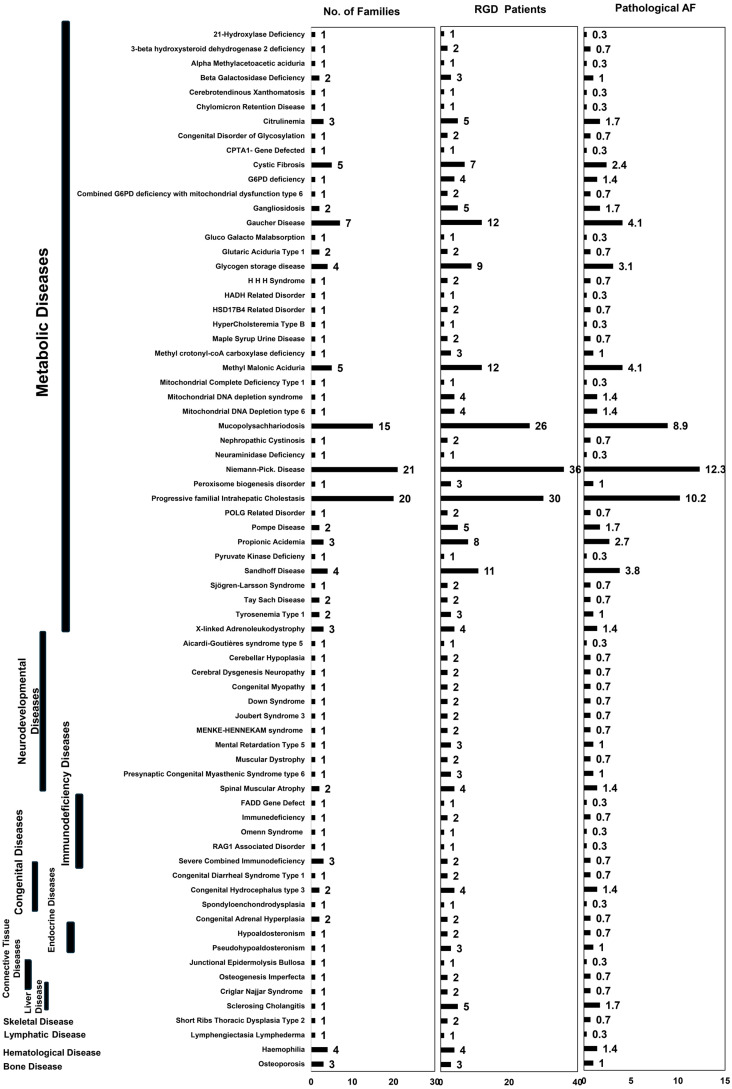
Distribution of RGDs among studied families. Left panel shows the number of families affected by each RGD, highlighting the variable incidence across diseases. Middle panel presents the incidence of RGDs within the cohort, reflecting the overall disease burden in the registry. Right panel displays the pathological allele frequency within, indicating compound burden of RGD-causing allele.

Of these 72 RGDs, 42 were of metabolic origin, including 37 inborn errors of metabolism; 11 neurodevelopmental diseases; five immunodeficiency diseases; three congenital, endocrine or connective tissue diseases; and one lymphatic, skeletal, hematological, or bone disease. In our registry, we had 21 families with Niemann-Pick (NP), 20 with PFIC [including PFIC I, PFIC II and PFIC IV], 15 with MPS [including MPS I, MPS IIIA, MPS IIIB, and MPS IV], seven with Gaucher disease, five with methyl malonic aciduria or cystic fibrosis, four with glycogen storage disease, hemophilia, or Sandhoff disease, three with X-linked adrenoleukodystrophy, severe combined immunodeficiency (SCID), osteoporosis, propionic acidemia, gangliosidosis, or citrullinemia, two with congenital adrenal hyperplasia, congenital hydrocephalus type 3, glutaric aciduria, Pompe disease, spinal muscular atrophy, Tay Sach disease, or tyrosinemia. For the remaining RGDs in the registry, only a single family was registered per RGD. We used single common phenotypic name for RGDs with more than one phenotype reported including PFIC and MPS. The number of RGD carrier families is shown in Figure 1. In our registry, 36 of the 72 RGDs were categorized as ultra-rare diseases according to the global RGD directories (Supplementary Figure S2). No hyper-rare disease was found in the current cohort. Two RGDs, PFIC and gangliosidosis, known as ultra-rare diseases globally, were not rare in our registry, with 20 and three carrier families, respectively. Also, we reported gangliosidosis and its subtypes (Sandhoff disease and β-galactosidase deficiency) separately based on their differing genotypes and phenotypes. In addition, the five most common diseases, NP, PFIC, MPS, Gaucher disease, and methyl-malonic aciduria, contributed to approximately 40% of the burden in this registry.

Of the 91 primary variants, 76 were SNVs, six were deletions, five were insertions, three were CNVs, and one was an inversion. Among the 58 secondary variants, 45 were SNVs, 11 were deletions, one was an insertion, and one was a substitution. We examined the variant repertoire of the three most common RGDs. All NP families have a pathogenic mutation in the *SMPD1* gene, inducing defective sphingomyelin metabolism, leading to visceral lipid accumulation. All patients with NP had abdominal distention, hepatomegaly, splenomegaly, and failure to thrive. Other variable phenotypes included abnormal bone marrow morphology, anemia, thrombocytopenia, elevated liver transaminases, and lower respiratory tract infections. All infants were diagnosed with NP within the first 12 months of life. While looking into MPS, we found that all MPS I patients had pathogenic mutations in the *IDUA* gene, mutations in the *SGSH* gene in MPS IIIA, mutations in the *NAGLU*, *GNPTAB*, and *HFE* genes in MPS IIIB, and mutations in the *GALNS* gene in MPS IV. Coarse facial features were observed in all MPS patients with other variable symptoms, including macrocephaly, hepatomegaly, short stature, and global developmental delay. All patients were diagnosed with MPS within the first 42 months of life. Among patients with PFIC, PFIC I was caused by a mutation in the *ATP8B1* gene, PFIC II by mutations in the *ABCB11* gene, and PFIC IV by mutations in the *TJP2* gene. All patients had only a primary variant in PFIC, except for three families with PFIC II and one family with PFIC IV. Among the three PFIC II families, the first family had secondary pathogenic variants in the *DPYD*, *G6PD*, and *PYGL* genes. The second PFIC II family also had a secondary variant in the *G6PD* gene. The third PFIC II family had one secondary variant in the *ABCB11* gene and another in the *KCNJ1* gene. All these patients with PFIC had hepatomegaly, elevated liver transaminases, and hyperbilirubinemia, while a variable picture of other symptoms including cholestasis, coagulation abnormalities, pruritus, seizures, and developmental delay, and were diagnosed within the first 18 months of life.

The current registry is naïve and is still recruiting patients in collaboration with clinicians, hospitals, and PTGDPRI’s province-wide district centres. To date, a snowball sampling strategy has been used, and our registry is not representative of population-wide RGD statistics. However, this is the first study to identify the incidence of many RGDs in the region. Thus, we did not use any population-wide statistics to report RGD incidence but the number of patients and pathological allele frequencies (AF) within the registry. We have shown the number of patients per RGD in Figure 1, which illustrates that NP has the highest incidence in our registry, followed by PFIC and then MPS. This incidence is representative of the accumulated burden of affected children with each disease. We calculated the pathological allele frequency of each registered RGD in our registry, as shown in Figure 1. We counted the zygosity of RGD carriers and affected children in this registry and calculated the pathological allele frequency for each RGD. The pathological allele frequencies in our dataset ranged from 0.34% to 5.80%. The highest number of pathological alleles were observed in NP (12.3%), PFIC (10.2%), and MPS (8.9%) in our registry.

### 2.2. Epidemiological Profiling of RGD Carrier Families

**Figure 2:**
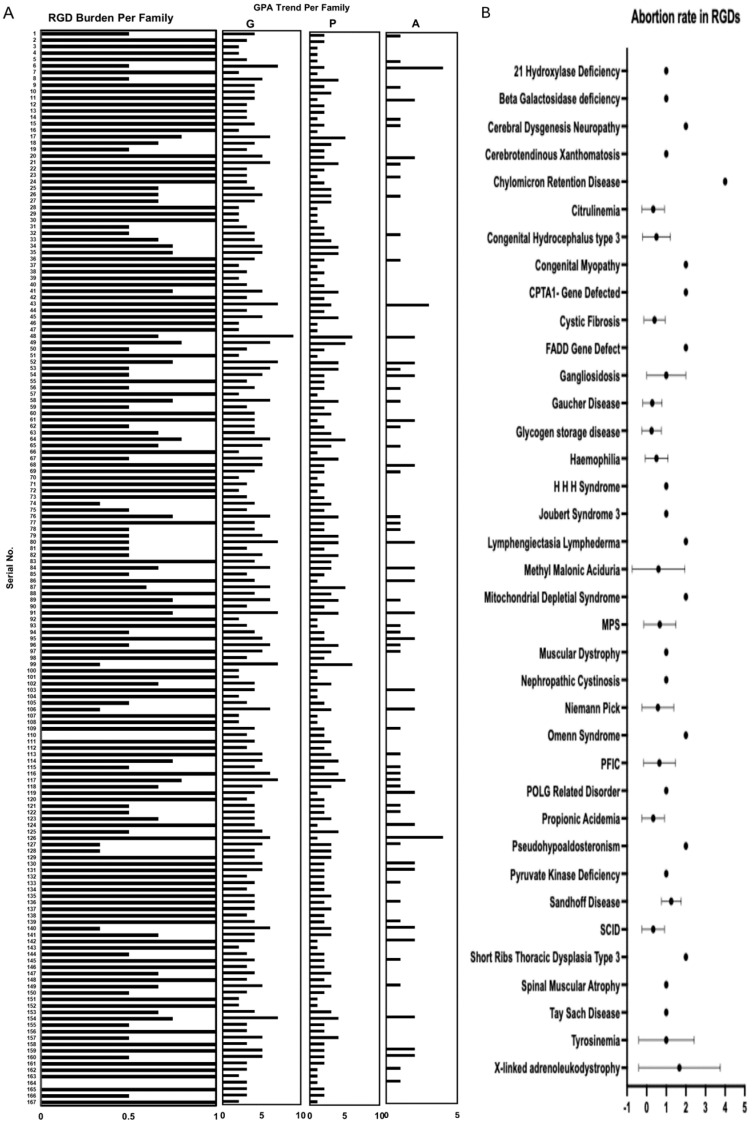
GPA profile and abortion rate among RGDs families. (**A**): Family wise GPA profile among RGDs families is shown. This analysis highlights the variations in reproductive history and pregnancy outcomes within affected families. (**B**): Distribution of abortion rate by RGDs is shown indicating coincidence of specific RGDs and abortion.

In total, 167 families were included in our dataset, with 293 affected children. We extended our search criteria and counted the total number of children including probands, children who died, and unaffected children at the time of registration. On average, each family had 2.34 ± 1.1 children per family. Of these, 0.71 ± 0.6 were diagnosed with RGD. These families also reported that their 1.13 ± 1.1 children died due to an RGD. Ultimately, these families had only 0.59 ± 0.7 normal children per family. The total number of children per couple (2.34 ± 1.1) in this registry was lower than the national average of 3.6 per couple [61], suggesting lower fertility in RGD carrier families. Of the 391 children, 188 died, with a mortality rate of 480 children per 1000, which is significantly higher than the national child mortality rate (62/1000) [61]. These statistics of higher mortality and fewer normal children in RGD carrier families are indicators of poor quality of family life. The burden of RGD per family (0–1) was calculated as the ratio between affected and total children in each RGD family. The overall burden of RGD children per family is presented in Figure 2A. Mean burden of affected children among all families was 0.81 ± 0.24. The maximum RGD burden observed was 1.0 in 95 families, which means that all born children were affected by an RGD. However, the lowest disease burden was 0.3, observed in six families with a history of RGD carriers who walked into PTGDPRI for prenatal screening and genetic counselling.

The family wise plot of self-reported gravida (G [total pregnancies]), parity (P [viable births]), and abortions (A), collectively termed GPA, revealed important reproductive health patterns (Figure 2A). The G per family generally ranged between one and nine (mean 3.92 ± 1.4), with P between one and six (mean 2.34 ± 1.1), and A between one and four (mean 0.58 ± 0.8). Most of these families were registered while they were pregnant, and the outcome as P or A was not yet understood. The close observation of P with G suggests consistently lower P underscoring the impact of A. These findings highlight the general reproductive trends in the cohort, with high desired fertility rates (G) and moderate outcomes as viable births (P) due to the influence of A. These insights are valuable for maternal health interventions and understanding reproductive patterns in RGD carrier families. These important findings suggest that the desired fertility rates are higher, but the actual fertility rates are lower among RGD carrier families.

We also identified 37 RGDs with co-incidence of spontaneous or elective abortion in our registry (Figure 2B). These 37 RGDs were observed in 66 families. Moreover, we observed that couples who are carriers of chylomicron retention disease or X-linked adrenoleukodystrophy are at a higher risk of having an abortion. Although we only had one family with chylomicron retention disease, this family had a history of four abortions. Chylomicron retention disease is a life-threatening disease caused by a rare autosomal mutation in the SAR1B gene, and patients cannot absorb fats or fat-soluble vitamins due to the lack of chylomicron synthesis and secretion. This is family 6, as shown in Figure 2A, had a G:P:A of 7:2:4, which included one current pregnancy with four abortions, and only two fetuses survived. Among these two, only one was normal, and the other was a patient with chylomicron retention disease.

In addition to chylomicron retention disease, X-linked adrenoleukodystrophy also showed the highest abortion rate. Three families with X-linked adrenoleukodystrophy were included in our registry (families 125, 126, and 127 in Figure 2A). These families had G:P:A ratios of 5:4:0, 6:1:4, and 5:3:1. Family 125 did not have any abortions, but family 126 had the highest abortion incidence of four fetuses. Family 127 had six pregnancies, including the current pregnancy, but only one fetus survived and was diagnosed with X-linked adrenoleukodystrophy.

### 2.3. RGD Burden Across Ethnic Groups

**Figure 3:**
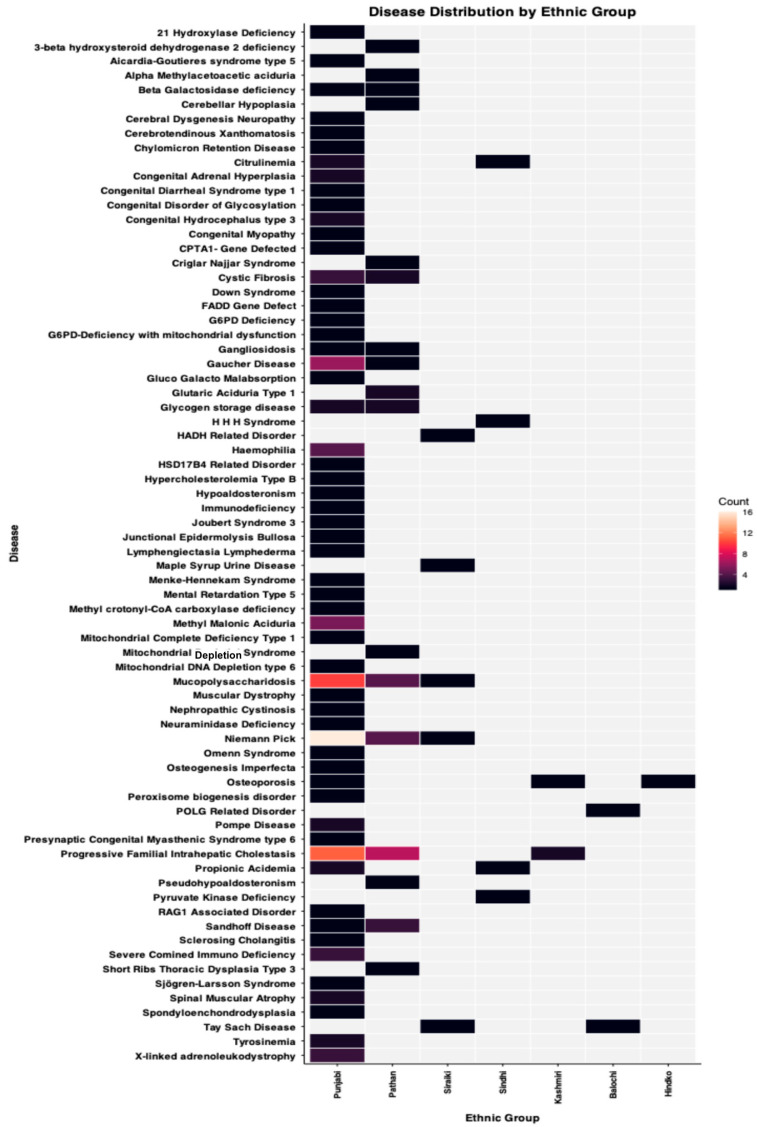
RGDs distribution among ethnic groups. RGDs are distributed among different ethnic groups, highlighting differences in the frequency of RGD within each group.

Although our institute is based in Punjab, RGD patients of both Punjabi and non-Punjabi ethnic origins are part of our registry. These non-Punjabi ethnic groups are either residents of the province of Punjab or registered with the PTGDPRI for clinical services from other provinces. According to ethnicity, there were 118 RGD families of Punjabi ethnic origin, 34 Pathan, five Saraiki, four Sindhi, three Kashmiri, two Balochi, and one Hindko. Our data show clear patterns of differential distribution of RGDs in ethnic groups that emphasize the importance of inclusion in RGD studies. These trends were most significant when comparing the Punjabi population with all other populations, as demonstrated in Figure 3. The Punjabi population demonstrated a significantly higher incidence of RGDs than the non-Punjabi population. A close examination of Figure 3 shows that 58 RGDs are present in the Punjabi population in our registry. Of these 58 RGDs, 47 were specific to the Punjabi population in the current registry, indicating a possible founder effect or a higher prevalence of pathogenic alleles within the population.

While looking into the non-Punjabi population, we found 16 RGDs present in the Pathan population. Of these 16 RGDs, eight were present only in the Pathan population. These eight RGDs include 3-β hydroxysteroid dehydrogenase 2 deficiency, α-methyl acetoacetic aciduria, cerebellar hypoplasia, Crigler-Najjar syndrome, glutaric aciduria type I, mitochondrial depletion syndrome, pseudohypoaldosteronism, and short rib thoracic dysplasia type 2. Five RGDs, including cystic fibrosis, gangliosidosis, Gaucher disease, glycogen storage disease, and Sandhoff disease, were cumulatively present in the Punjabi and Pathan populations. The other three diseases (NP, MPS, and PFIC) were shared by more than two ethnic groups.

Five RGDs were present in the Saraiki population. Of these five, two RGDs (HADH-related disorder and Maple Syrup Urine Disease) were only present in Saraiki ethnic families. Four RGDs were observed in the Sindhi population. Among these RGDs, two (HHH Syndrome and Pyruvate Kinase Deficiency) were specific to the Sindhi population in this registry. Moreover, two other RGDs (citrullinemia and propionic acidemia) were shared between the Punjabi and Sindhi populations. Among the two Balochi ethnic families, the POLG-related disorder was observed in one family and was specific to the Balochi population. However, Tay-Sachs disease, the second RGD observed in the Balochi ethnic group, was also found in the Saraiki population, consistent with the colocalization of both groups in the Southern Punjab region. In the Hindko ethnic group, we observed only one disease, osteoporosis, which was also shared among the Punjabi and Kashmiri ethnic groups, consistent with the historical closer geographic existence of the Hindko, Kashmiri, and Punjabi populations. Though several RGDs show specificity towards a specific ethnic group but it may not be a true representativeness due to lower sample sizes and snowballing, but it should be considered as recorded incidence of particular RGDs in those ethnic groups.

### 2.4. RGD Distribution Across Castes

**Figure 4:**
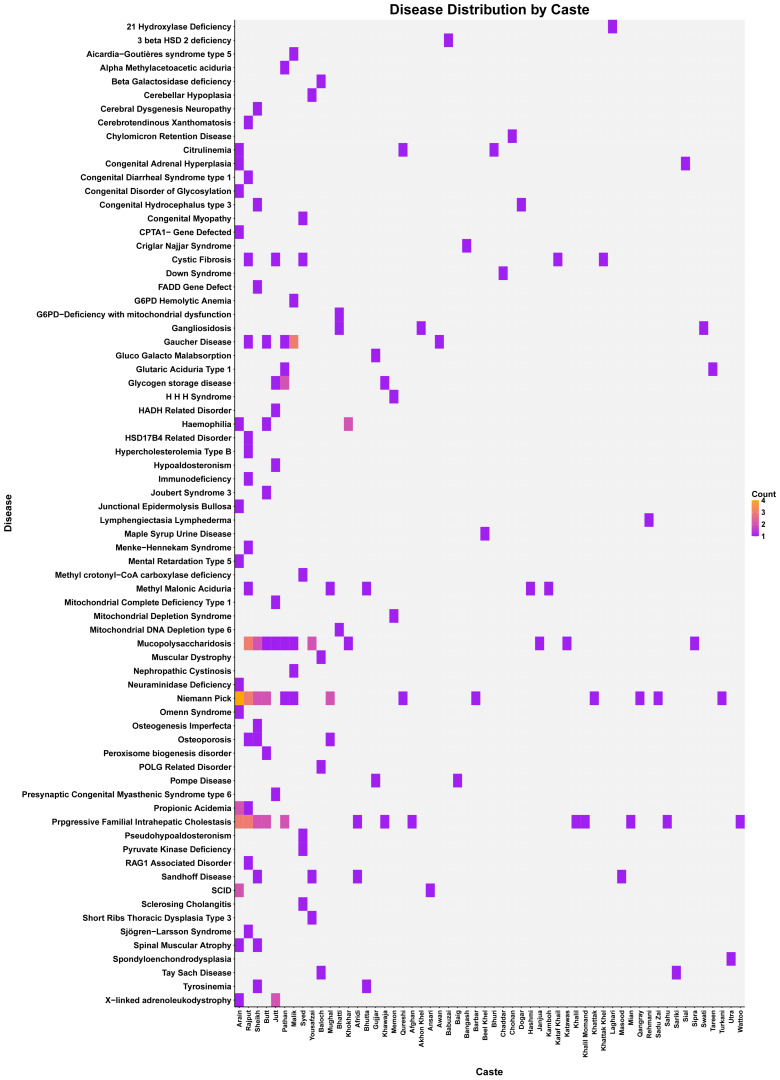
RGDs distribution among castes. Distribution of RGDs by castes is shown. It demonstrates how the occurrence of specific disease varies among castes reflecting underlying genetic patterns.

Ethnicity was further subdivided into castes. The number of families carrying an RGD within each caste is shown in Figure 4. In Punjabi castes, highest RGD incidence was recorded in Arain (22, 13.2%) and Rajput (22, 13.2%), followed by Sheikh (14, 8.4%), Butt (9, 5.4%), and Jutt (9, 5.4%) castes. On a disease-wise pattern, we found that 21 RGDs were distributed across the castes and 51 RGDs were specific to only one caste, while others, including MPS, NP, osteoporosis, and PFIC, were distributed across the castes. Among Rajput caste, seven RGDs were caste-specific including Cerebrotendinous Xanthomatosis, congenital diarrheal syndrome, HSD17B4-related disorder, hypercholesterolemia type B, Menke-Hennekam Syndrome, RAG1-associated disorder and Sjögren-Larsson Syndrome. Six RGDs were specific to the Arain caste, including congenital disorder of glycosylation, CPTA1-gene defect, junctional epidermolysis bullosa, mental retardation type 5, neuraminidase deficiency, and Omenn syndrome. Five RGDs were specific to Sheikh caste, including cerebral dysgenesis neuropathy, FADD gene defect, osteogenesis imperfecta, spinal muscular atrophy, and tyrosinemia. The Jutt caste had three caste-specific RGDs, including hypoaldosteronism, mitochondrial complete deficiency, and presynaptic congenital myasthenic syndrome. Among the Butt caste, two RGDs (Joubert syndrome and peroxisome biogenesis disorder) were caste-specific.

Looking deeper into caste-specific disease incidence, we found that most castes had a specific RGD incidence, except Arain, Baloch, Barber, Bhatti, Butt, Gujjar, Jutt, Khawaja, Khokhar, Malik, Memon, Mughal, Pathan, Qureshi, Rajput, Sheikh, Syed, and Yousafzai. All other castes showed a specific incidence of RGD; however, an epidemiological sample size-aware survey is needed to understand if RGDs are caste-specific or identified by chance. A detailed outlook of the RGD-caste relationship is shown in Figure 4.

### 2.5. Association of Consanguinity with RGDs

There were four categories for recording the consanguinity status of the parents in RGD carrier families: first cousin, second cousin, relative, and unrelated. Among the RGD families, first cousin marriages were 129 (77.2%), second cousin marriages were 14 (8.4%), marriages within relatives were 7 (4.2%), and 17 (10.2%) couples were married to an unrelated spouse. Cumulatively, first cousins, second cousins, and marriages within relatives accounted for 89.8% of the current cohort in this registry. This is one of the highest consanguinity rates observed in RGD carrier families.

**Figure 5:**
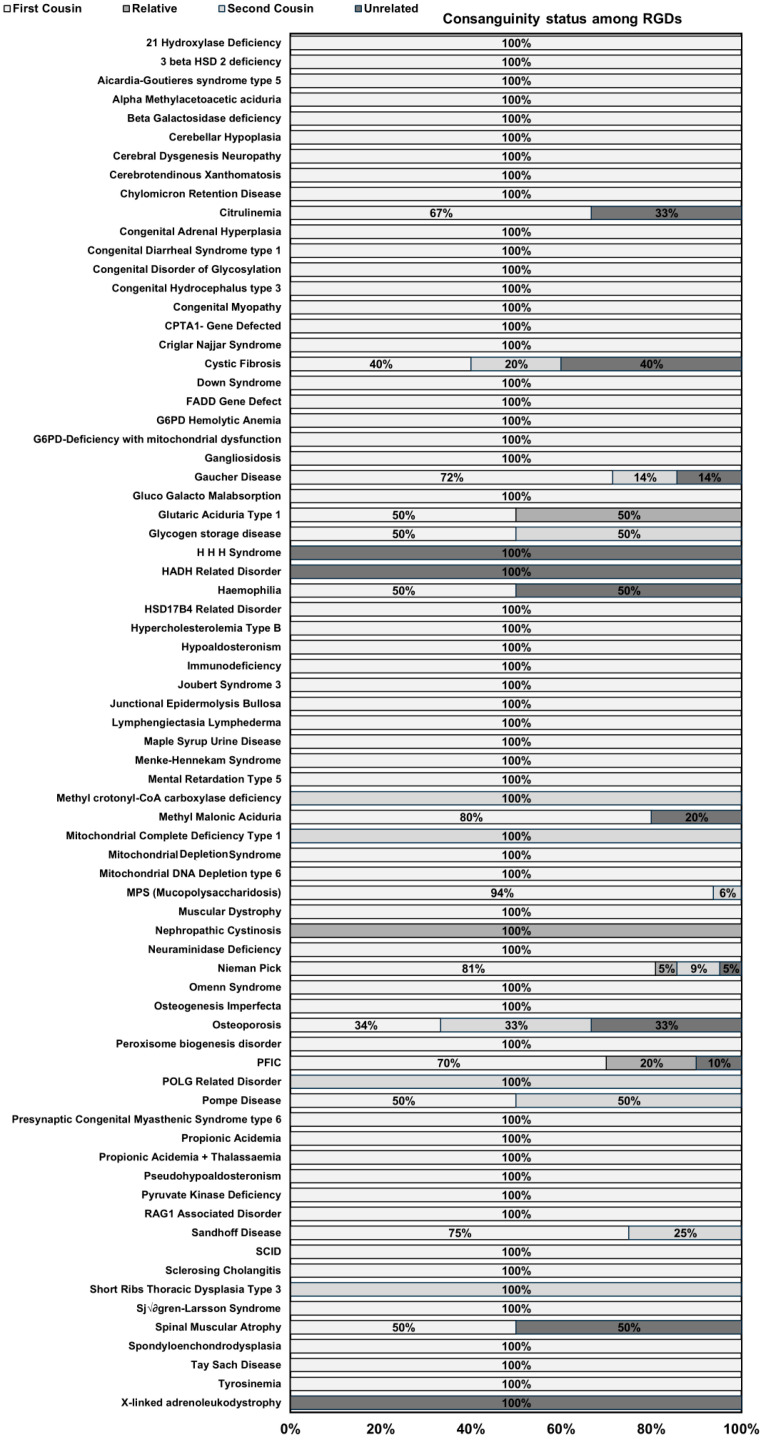
Consanguinity comparison among RGDs. Consanguinity status among RGD carrier families is presented. Consanguinity is recorded as first cousin marriage, second cousin marriage, marriage with a relative and marriage with unrelated spouse.

To illustrate the distribution of families affected by various RGDs according to consanguinity status, a stacked bar plot shows RGD-wise consanguinity rates in Figure 5. The visualization in Figure 5 highlights a pronounced trend in which a significant number of RGDs showcase consanguineous marriages, particularly first-cousin marriages. In our current registry cohort, 100% first-cousin marriages were observed in 50 RGDs, 100% second-cousin marriages were observed in four RGDs (methyl crotonyl-coA carboxylase deficiency, mitochondrial complete deficiency type 1, POLG-related disorder, and short rib thoracic dysplasia type 2), 100% marriage with a relative in one RGD (nephropathic cystinosis), and 100% marriage with an unrelated person in three RGDs (HHH syndrome, HADH-related disorder, and X-linked adrenoleukodystrophy). Thus, 69 out of the 72 RGDs in our registry showed a history of consanguinity, suggesting an increased risk associated with the incidence of RGD in this cohort. A total of 14 RGDs showed mixed patterns of consanguinity. These RGDs include cystic fibrosis, citrullinemia, Gaucher disease, glutaric aciduria type 1, glycogen storage diseases, hemophilia, methylmalonic aciduria, MPS, NP, osteoporosis, PFIC, Pompe disease, Sandhoff disease, and spinal muscular atrophy. All these RGDs showed different percentages of consanguinity patterns, but the consanguinity rate remained 50% or higher, consistent with our observation of consanguinity as a risk factor. To further assess the statistical association between consanguinity and RGDs, we used previously published randomly collected consanguinity data of 1011 families from the Punjab Consanguinity Survey with no RGDs [61] and compared them with our consanguinity records using a chi-square test (X^2^ = 44.78, *p*-value < 0.00001). The association was extremely statistically significant, supporting our observation of the risk association of consanguinity with RGDs. To further understand the direction of this association, we used several effect size measures, including the odds ratio (OR), reciprocal of OR, relative risk (RR), and reciprocal of RR. The OR showed five times higher odds of consanguinity (OR = 5.05, 95% CI 2.999–8.395) in RGD families in this study, while the reciprocal of OR showed a lower probability (reciprocal OR = 0.198, 95% CI 0.1191–0.3335) of non-consanguinity incidence in RGD families. Together, these statistics confirm the statistical association of consanguinity as an associated risk factor to RGDs. The RR showed more than fourfold higher consanguinity (RR = 4.28, 95% CI 2.66–6.96) in RGD carrier families than in non-RGD healthy controls, with reciprocal RR showing that non-consanguinity is rare (reciprocal RR = 0.2334, 95% CI 0.1437–0.3761) among RGD carrier families.

Significant variations in consanguinity patterns among different ethnic groups were also observed, and the cumulative share of each ethnic group is shown in Supplementary Figure S3. Punjabi, Pathan, Saraiki, and Sindhi shared both consanguinity and non-consanguinity patterns, while Kashmiri, Balochi, and Hindko showed only consanguinity patterns.

### 2.6. Molecular Epidemiology of RGDs in Current Registry

Following the registration and identification of RGDs in the current registry, we examined genetic variants identified by NGS in 91 RGD families spanning a repertoire of 109 genes, including primary and secondary RGD findings. Most RGDs and causal genes are unique to each family; however, there are RGDs in which the causal gene or pathogenic variant is observed in more than one family. The family-wise incidence of each RGD and gene repertoire is shown in Supplementary Table S2A. These 109 genes cumulatively had 131 variants, and disease-associated genes and variants with family incidence are listed in Supplementary Table S2B. The gene-wise relationship is presented in Supplementary Table S2C, while the variant-wise relationship is presented in Supplementary Table S2D.

**Figure 6:**
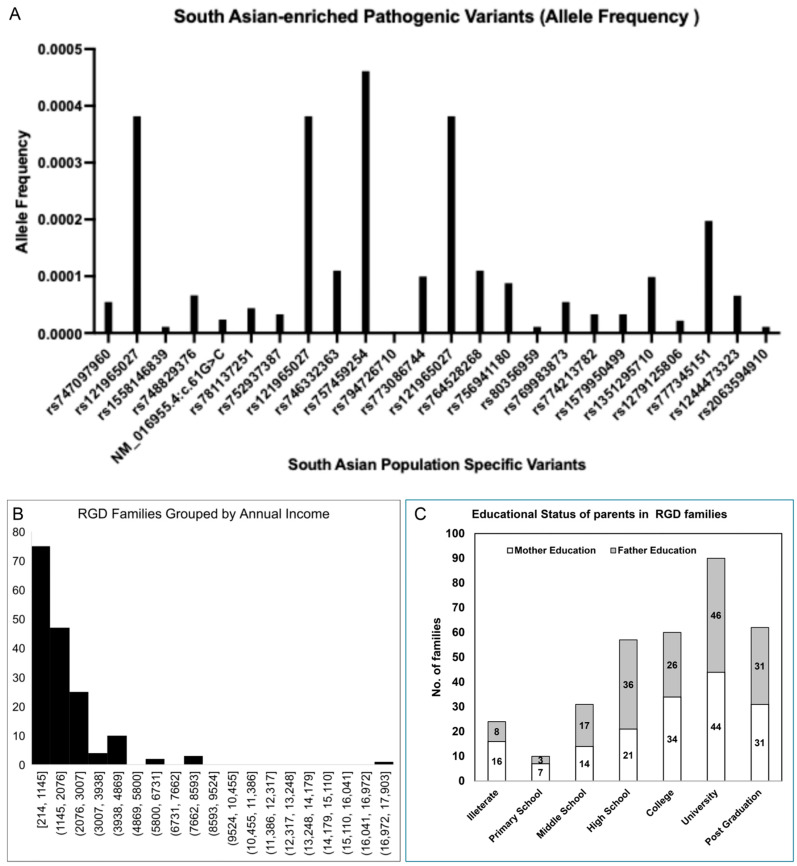
Highlights of demographics and South Asian-enriched pathogenic variation in study cohort. (**A**): Allele frequency of South Asian-enriched variants identified in current study. (**B**): Annual income of RGDs families in current study plotted in eight groups dividing low-, low-middle-, high-middle- and high-income groups. (**C**): Educational status of RGD parents in current study exhibits major proportion of highly literate parents.

For each of the 131 variants, we annotated rsIDs and allele frequencies from the database of single nucleotide polymorphisms (dbSNP) and genome aggregation database (gnomAD). These allele frequencies, along with rsIDs, are listed in Supplementary Table S2A. The allele frequencies in this table are categorized according to global ethnicities, including African, American, European, Middle Eastern, and South Asian ancestries. The purpose of this comparison was to determine whether the pathogenic variants in our dataset are population-specific or globally distributed. Although most pathogenic variants from our cohort were global, 24 pathogenic variants associated with 23 RGDs were exclusively reported in the South Asian population and were absent in other global ancestries. This population specificity of pathogenic variants of associated RGDs argues whether these variants were founded in the South Asian population; however, due to the small data size and methodological limitations, we could not confirm founder events and termed them South Asian-enriched pathogenic variants. The allele frequencies of these variants, according to the gnomAD database, are shown in Figure 6A. These 23 RGDs with South Asian-enriched variants include Aicardi-Goutières syndrome type 5, α-methyl acetoacetic aciduria, aspartyl glucosaminuria, breast cancer, Cerebrotendinous Xanthomatosis, congenital hypoaldosteronism, dihydro-pyrimidine-dehydrogenase deficiency, hemophagocytic lymphohistiocytosis, junctional epidermolysis bullosa, microencephaly, MPS I, MPS IV, neuraminidase deficiency, Omenn syndrome, ornithine transcarbamylase deficiency, PFIC II, Pompe disease, pontocerebellar hypoplasia type II, Sandhoff disease, severe combined immunodeficiency, spondyloenchondrodysplasia, Stromme syndrome and 3-β hydroxysteroid dehydrogenase type II deficiency. Among these diseases, the same MPS I South Asian-enriched pathogenic variant in the IDUA gene was observed in three different families.

In addition to the South Asian population specificity, we investigated variants with shared origins in the European and Middle Eastern populations. We found 19 variants with ancestry shared between South Asian and non-Finnish European populations only (Supplementary Table S2A). Moreover, we found two variants of shared ancestry between the South Asian and Middle Eastern populations according to the gnomAD allele frequency database. Four variants were shared among South Asian, European, and Middle Eastern populations. The higher shared variants observation between South Asian, Middle Eastern and European populations is consistent with Out-of-Africa migration history. It is also worth noting that both the current study population and the European population have Caucasian origins. Identification of South Asian-enriched pathogenic variants in this study is also suggestive of inclusion of underrepresented populations in global genomic and clinical projects.

### 2.7. Association of Socioeconomic Demographics with RGDs

The entire family of RGD patients is affected psychologically, socially, culturally, and economically. In this study, we conducted an in-depth evaluation of RGD carrier families to investigate whether they belonged to low-, middle-, or high-income groups. In the current cohort of our registry, the annual income of RGD carrier families ranged between $214 and $17,142, with a mean annual income of $1780. An income group classification is shown in Figure 6B, where RGD families are divided into eight groups with an interval size of 931. We used the World Bank’s gross national income (GNI) per capita as the annual income per RGD carrier family to rank the economic status of each family. The first group, with an annual income between $214 and $1145, had 75 RGD carrier families, which is consistent with the World Bank’s low-income group, suggesting a high incidence of low-income families carrying RGDs. Second, there were 86 families with annual family incomes between $1145 and $4869, consistent with the World Bank’s low-middle income group. The third group, with an annual family income between $5800 and $8593, ranked as the high-middle income group, and had five RGD carrier families. The fourth group had only one family with an annual income of $17,142 and ranked in the high-income group. In conclusion, the 161 families in the current cohort belonged to either the low- or lower-middle-income groups, implying a higher economic burden on the RGD carrier families. The World Bank ranks Pakistan as a low-middle income country, but in our cohort, 45% (75 RGD families) of families had an income lower than the national bar, implying the worst economic pressure. While assuming a higher economic burden, it is necessary to remember that there is no national health insurance system in Pakistan; therefore, any costs associated with the patient are out-of-pocket expenses for the RGD family, creating inequities in accessing standard healthcare.

We further collected data on the literacy rate, including the highest education status of these 167 RGD families. Figure 6C shows the educational status among RGD families, with the highest number of spouses with at least university-level education, followed by post-graduation, college, and high school level education. This high literacy rate in this cohort is consistent with the growing literacy rate in Pakistan and provides a niche for genetic counselling and better care of RGD patients if appropriate resources are provided at the national or provincial scale.

## 3. Discussion

This study is the first and only RGD registry with molecular, health, and socioeconomic data from Pakistan. In this manuscript, we report the spectrum of RGDs in the Punjabi population for the first time. Several individual case reports of RGDs in the Punjabi population have been published; however, a population-wide spectrum is not known. The PTGDPRI took the initiative to build a province-wide registry and registered 167 families with 72 RGDs of pediatric onset (Figure 1). These 72 RGDs include metabolic diseases, neurodevelopmental diseases, immunodeficiency diseases, connective tissue diseases, and others. In addition to disease identification, we annotated 109 genes with 106 RGD phenotypes, including primary and secondary diagnoses. These genes showed 91 primary variants and 58 secondary variants, of which 31 variants among these were VUS. Furthermore, we identified RGDs among Punjabi and non-Punjabi ethnic groups, and a caste-wide incidence was also presented. In our registry, we also incorporated consanguinity as a risk factor for RGDs and found that 89.8% of RGD carrier families were consanguineous, and an association between RGD and consanguinity was observed. Molecular epidemiology methods suggested that 24 of the total registered variants were specific to South Asian populations and were annotated as the South Asian-enriched pathogenic variants. Finally, we observed an economic burden giving rise to inequities in accessing healthcare, despite a high literacy rate among registered RGD families.

As mentioned earlier, PTGDPRI, a provincial government institute known for genetic testing of thalassemia and other genetic diseases, came across the line of action to prevent such RGDs that are life-threatening and affect a large population of the Punjab region. In the dataset presented in this manuscript, 167 RGD carrier families were registered in PTGDPRI and obtained clinical services, including CVS, prenatal genetic testing, and genetic counselling, based on their clinical picture or past clinical history; thus, the majority of registered families were already identified as risk groups. In the current dataset, registered families had 293 affected children, including 188 who had already died, and only 98 children were born normal. We have calculated burden of RGD-affected children in these families and mean RGD-burden was 0.81 ± 0.24 out of 1.00 presenting a significant RGD-affected children burden on each family (Figure 2A). Although several previous studies have reported the incidence of genetic diseases in the Pakistani and Punjabi populations, this evidence remains generalized to phenotypes, traits, and common genetic diseases such as hemoglobinopathies [62,63,64,65,66,67]. In addition to these traits and common genetic diseases, there have been many case reports of RGDs, but collective evidence of a population-wide spectrum is lacking. This is the first report of clinically diagnosed RGDs based on molecular evidence. RGD patients are mostly children, with associated higher morbidity and mortality, and the worst of these is the diagnostic odyssey [68]. The RGD carrier families in our registry represent the final outcome of any diagnostic odyssey they faced in the past. Despite the availability of this comprehensive RGD dataset, we did not calculate any epidemiological measures, such as prevalence, as data were collected through snowball sampling, and such calculations could induce a non-random bias. Instead, we counted the alleles of all causal genotypes together and calculated the cumulative pathological allele frequency to represent the RGD share in this registry. This registry not only compiles epidemiological information on RGDs but also serves as a platform to introduce carrier families to their desired clinical services through its field presence in the Province of Punjab.

In our registry, we found that metabolic RGDs were the most prevalent, followed by neurodevelopmental, immunodeficiency, congenital, endocrine, connective tissue, lymphatic, skeletal, bone, and hematological RGDs. This is the first population-based evidence to understand RGD trends. Among the high-incidence metabolic RGDs, 37 were inborn errors of metabolism. Although these metabolic RGDs, including inborn errors of metabolism, are fatal, many of them are curable after the identification of metabolic malfunction or metabolic element deficiency [69]. This potential cure highlights the need for fast and accurate genetic testing availability to the general population and/or at least to carrier families which is currently a challenge for RGD carrier families. Unfortunately, there are no provincial or national pathways available for the diagnosis and cure of RGDs in Punjab or Pakistan. This reduced access to diagnosis and treatment gives rise to clinical complications in patients and alarms governments to develop appropriate policy for accessible and affordable healthcare. Although we did not register it as a variable however patient interviews revealed that none of the patients were under appropriate treatment but symptomatic. Factors contributing to inequity in accessing appropriate treatment include a lack of specialized clinical services, trained clinical geneticists, physicians, and molecular pathologists, and the unavailability of both clinical testing and treatment options. These facilities and resources can only be built at public discretion, but they remain ignored.

In this study, the five most common RGDs were NP, PFIC, MPS, Gaucher disease, and methyl malonic aciduria. Together, these diseases account for approximately 40% of the RGD burden in our registry. In addition to the higher incidence of RGDs, we also observed varying phenotypes of PFIC and MPS. These phenotypes included PFIC I, PFIC II, and PFIC IV for PFIC, and MPS I, MPS IIIA, MPS IIIB, and MPS IV for MPS. In addition to these RGDs, many other families exhibit several secondary variants in addition to the primary variants. The existence of secondary variants is evidence of the unique genotype of each RGD patient, which then produces a unique phenotype of the clinical condition. In addition, these molecular findings of unique genotypes remain consistent for associated primary and secondary genes and pathways; thus, a pathway-based approach can also be established for the identification of RGDs at the molecular level. Current diagnostic best practices mainly include WGS, WES, or targeted-capture sequencing, which are not always successful because of the complexities associated with RGDs and the technical limitations of these technologies. However, the involvement of multiple genes suggests that the disruption of physiological pathways can be used in a reverse fashion to identify associated genes or at least understand the molecular-scale pathology of RGDs. The PTGDPRI currently only offers prenatal testing and genetic counselling, but a complete solution with intervention is desired by provincial or national stakeholders. Several treatment regimens with regulatory approval are already available for some of these RGDs, and immediate relief in terms of accessible and equitable clinical services is necessary [70,71,72,73,74,75]. In addition to the available treatment regimens, many potential therapeutics are being developed and undergoing clinical trials. Access to potential therapeutics for these patients is also necessary for clinical trial enrollment.

Furthermore, it is not only about RGDs; we attempted to incorporate the maximum available information associated with RGDs. The impact of carrying RGD-associated alleles does not only affect newborn children but also worsens the family well-being and reproductive health of carrier mothers, as shown by higher child mortality and GPA statistics in our registry. We learned that the families in our registry had 2.34 ± 1.1 total children, with only 0.59 ± 0.7 normal children per family. The remaining children were either affected or deceased. The mean mortality burden per family in our registry was 1.13 ± 1.1, which is approximately half of the total mean number of children in each family. These data suggest a child mortality rate of 480 per 1000, which is significantly higher than the national average of 62 children per 1000, arguing that higher mortality rates (seven times the national average) are associated with RGDs [61,76]. These higher numbers of mortality and RGD-affected children worsened family well-being, although we did not investigate this on an established scale. In addition to higher mortality, RGD carrier mothers also face a higher incidence of abortion. The GPA statistics (Figure 2A) indicated that the mean G was 3.92, with mean P, 2.34 and mean A, 0.58. This P is also representative of the fertility rate among RGD carrier mothers, which is much lower than the national fertility rate of 3.6 per mother [61,76], arguing for lower fertility rates than the national average. The difference between P and the other two statistics, G and A, represents the fertility challenges faced by RGD carrier mothers and families, especially in terms of lower viable births and higher rate of abortions. The G in our dataset is slightly higher than the national fertility rate of 3.6, indicating a higher desired fertility trend. Although this trend of abortions was not common among all RGDs, 37 RGDs showed an incidence of A in 66 RGD families (Figure 2B). In Pakistan, several maternal health surveys have revealed population rates of A between 29 and 66 per 1000 women [77,78,79]. However, we believe that our current dataset is still too small to draw conclusions about the abortion rate in RGDs in the study population. However, based on our results, it is evident that at least 37 RGDs were observed together with an abortion event, and 66 carrier mothers out of 167 (395 per 1000 women, at least six times the national rate) had experienced an abortion at least once (Figure 2). Our study populations are notorious for cultural underreporting of abortions, but the observed abortion rate is still too high, setting a minimum abortion rate bar in RGDs.

The Punjabi population and other ethnicities living in Pakistan are anthropologically and genetically heterogeneous [80,81,82], representing historical migrations to this region [83,84,85,86]. Therefore, we profiled RGDs according to the major ethnic groups represented in our registry. These groups include Punjabi, Pathan, Saraiki, Sindhi, Kashmiri, Balochi, and Hindko families. Although all families were recruited from Pakistan, these ethnic groups are also widely distributed across other South Asian countries. The Punjabi population resides primarily in the Punjab province of Pakistan and in the adjoining Indian state of Punjab. The Pathan (Pashtun) population mainly inhabits the northwestern province of Khyber Pakhtunkhwa in Pakistan and the neighbouring regions of Afghanistan. The Saraiki population is concentrated in southern Punjab, Pakistan, and extends into the Indian Punjab. The Sindhi population lives in Pakistan’s Sindh province and the adjacent regions of Rajasthan and Gujrat in India. The Kashmiri people inhabit the Kashmir region, which is divided between India and Pakistan, while the Balochi population originates from Pakistan’s western province of Balochistan and extends into Iran and parts of Western and Central Asia. Thus, knowledge in this registry about ethnic groups is relevant not only to the Pakistani population but also to neighbouring populations in South, West, and Central Asia. In the current registry version, we have 118 families of Punjabi origin, 34 Pathan, five Saraiki, four Sindhi, three Kashmiri, two Balochi, and one Hindko. Among these registered families, 58 RGDs were observed, of which 47 were Punjabi population-specific. Among the registered Pathan families, 16 RGDs were present, of which eight were Pathan population-specific. Among the Saraiki-registered families, five RGDs were present, two of which were Saraiki population-specific. There were only two Balochi families in this registry exhibiting two RGDs, one of which was Balochi population-specific. However, the Hindko and Kashmiri populations shared RGDs with the Punjabi and Pathan populations, and no population-specific RGDs were identified in this registry. Although these findings highlight the population specificity of several RGDs, there is still a chance that these RGDs could occur in other ethnic population groups. Because these registry data are more observational and do not lead to the inference that other populations could not be affected by these traits unless a genomic survey of other populations for pathogenic variants is conducted. Thus, current registry data were used only to determine population-specificity within the registry and to identify specific RGD incidence in particular ethnicities; larger RGD cohorts should be studied to determine population-specificity.

To narrow down this ethnic analysis, we stratified the data in our registry by caste. Castes are anthropologically isonym groups that tell us the lineage of each individual and are used for identification purposes even in modern-day South Asian societies. In some non-Punjabi ethnicities, this lineage information is preserved with tribe names instead of castes. However, in this registry, we used both terminologies, caste and tribe, synonymously. Among castes, more than 50% of the RGD burden was found in five Punjabi castes (Arain (13.2%), Rajput (13.2%), Sheikh (8.4%), Butt (5.4%), and Jutt (5.4%)), and one non-Punjabi group (Pathan (5.4%)). Other castes with lower RGD incidence include Baloch, Bhatti, Malik and Syed. The castes with higher RGD incidence, including Arain, Rajput, and Jutt, are large caste groups widely present in the Pakistani and Indian Punjab regions, Sindh province of Pakistan, and Haryana region of India. Moreover, these castes also include tens of subcastes that are representative of each group’s anthropology, region, trade, or religion, while keeping the caste-wide history intact. Other castes, including Sheikh, Butt, and Pathan, are also widely present in Pakistan, India, present-day Kashmir, and Afghanistan, but their populations are smaller than the aforementioned castes. In our previous studies and other published literature, we learned that the incidence of several genetic traits was higher in Arain, Rajput, Jutt, and Sheikh castes [47,54,62,63,64,65,66,67]. Thus, our current registry is a valuable resource for caste-specific RGDs and could be useful for RGD screening of specific castes. In the current registry, we found that 51 RGDs were caste-specific. Rajput showed seven caste-specific RGDs, Arain six, Sheikh five, Jutt three, and Butt two caste-specific RGDs. However, these caste-specificity is still an observation in this registry and larger cohorts with statistically significant sample sizes should be used to make an inference. Our results on ethnicity and caste-specificity suggest the need to build a comprehensive registry of RGDs among South Asian ethnicities and castes. Such a registry could provide not only comprehensive information on ethnic and caste-specificity but also generate screening batteries for groups of RGDs. This is also a goal of the current registry to build a comprehensive resource and screening battery for a group of RGDs, which could be a quick and cost-efficient way to screen RGD-suspects before genetic testing and/or genomic profiling, especially in South Asian countries, where access to modern genetic technologies is still limited.

Following castes, we led our registry to another aspect of the social structure, consanguinity. In a society divided by castes, people prefer to interbreed within their caste as it is a matter of pride for their families. In addition, consanguinity is a practice to maintain social, political, and economic status within the family. Though a few religions in South Asia including Sikhism in Punjabi population prohibit consanguineous marriages but people practicing Islam vastly practice it not only in South Asian premises but also in Middle Eastern and Western and Central Asian regions [47,52,59,87,88,89,90,91]. In our registry, 69 out of 72 RGDs showed history of consanguinity practice (Figure 5). In our previous population-based surveys, we found incidence of consanguinity was over 80% in Punjab [47]. In the current registry, we observed 89.8% consanguinity in RGD carrier families, with 77.2% first cousin marriages. This is so far the highest consanguinity burden reported in any population in the region. As several previous studies have tested consanguinity as a risk factor for recessive genetic traits, so we have tested consanguinity status in our registry against non-RGD data available from Punjab Consanguinity Survey [61]. This analysis revealed a significant association between RGDs and consanguinity, and consanguinity remains the major contributor of RGDs in current RGD carrier families. Identification of RGD- and other lethal genetic trait-carriers pre-marriage can serve as a control strategy for RGDs inheritance to the next generation in this high consanguinity practice. The major contributors to consanguinity, as mentioned earlier, are social, economic and political reasons. So, we have investigated socioeconomic demographic factors including annual income and education status among RGD carrier families in this registry.

We found 75 RGD carrier families to be among low-income group, 86 among low-middle income groups, five among high-middle income and only one high income family (Figure 6). A recent study determined $5283 as mean yearly clinical costs associated with RGDs [92] while mean annual income in our registry was $1780 only. Though these costs were calculated from Canada and United Kingdom settings but these costs remain similar or higher in Pakistan due to lack of local laboratories and treatment access, and dependency on international supply chain of drugs and diagnostic services. Comparing our registry, it is impossible for low-income and low-middle income groups to afford these costs. Even for the high-middle income group, the mean annual income of families is similar to the mean yearly clinical costs, and it is nearly impossible to afford clinical costs for these families too, and only one high-income family can afford these costs. This suggests huge economic pressure and a lack of financial resources for the diagnosis and treatment of RGDs. Such economic pressures and long-standing diagnostic odysseys previously have been reported in Pakistani population, several developed nations and under-privileged populations [93,94,95]. This is also worth noting that unlike global north countries, no national health insurance or coverage system exists in Pakistan and all clinical costs are out-of-pocket ultimately an economic burden for these families despite existing economic pressure. Moreover, as we mentioned the mean yearly clinical costs of RGDs of $5283, we compared this cost of other clinical costs in Pakistan and learned mean out-door visit cost per patient is only $4.1 in a public setting [96], while the mean hospital stay cost in the case of an injured patient is $271 in a public setting and $451.7 in a private setting [97]. These comparisons suggest that the clinical costs of RGDs are approximately 11 times higher than those of regular patients and remain unaffordable in the absence of a national health insurance system, giving rise to inequity in access to clinical services. These data also suggest that in our current study population, the share of socioeconomic status and wealth may not be the primary reason for consanguinity but rather the lack of options for marriage partnerships due to economic stress. The Punjab Consanguinity Survey [61] also revealed similar outcomes, where the economic burden on families is among the major contributors to consanguinity practice in the Punjabi population. Despite the high economic pressure on RGD families, we found that the literacy rate among these RGD families was very high, which also reflects the socioeconomic dilemma of low wages and unemployment in Pakistan and other developing countries. However, in the current study, highly literate people are good resources for understanding RGDs and seeking genetic counselling.

Finally, we investigated the global allele frequencies of pathogenic variants in the current registry. The allele frequencies of pathogenic variants suggest the prevalence and incidence of particular genotypes and associated disease phenotypes. In our registry, we identified 131 variants in 109 genes associated with RGDs. We searched for their incidence among all major population groups and learned that 24 of these variants were of South Asian origin and were termed South Asian-enriched pathogenic variants. These 24 variants were associated with 23 RGDs, and these variants should be prioritized and incorporated into genetic tests and pan-genomic testing panels for RGDs for a fast, cheaper, and accurate diagnosis, minimizing the present odyssey. In addition, structural changes induced by these South Asian-enriched pathogenic variants should be addressed in ongoing novel drug discovery efforts and companion diagnostics. Due to small sample size and technical difficulties, we relied on allele frequency data at this time; however, future studies with large cohorts should be conducted to determine pathogenic haplotypes and their incidence within other South Asian and global populations. Such large cohorts would not only characterize population-specific variants but also will play a role in characterizing VUS. Lack of VUS characterization in understudied population at this moment makes expensive genetic testing inconclusive and a clinical challenge in genetic counselling of prenatal testing.

In addition to the direct results of our study, there are several indirect points of discussion, including the need for rapid and cost-efficient genetic testing and the need for provincial, national, and regional RGD support groups. The current registry is an effort by the PTGDPRI to understand RGDs dynamics, but the following efforts are needed by the provincial government of Punjab, the national ministry of health coordination, and the World Health Organization’s regional offices of the Eastern Mediterranean and Southeast Pacific regions. Pakistan is representative of a diverse, heterogeneous population with cultural roots shared between Southeast Asia and the Eastern Mediterranean Region. Several aspects reported in this study, including lower fertility rates among RGD carrier mothers, higher mortality rates among RGD carrier families, higher incidence of consanguinity in RGD carrier families, and identification of South Asian-enriched pathogenic variants, lay the foundation for policy-making in both regions. To better manage RGDs, national-level infrastructure, including newborn and prenatal screening of RGDs, is necessary and should be mandated as soon as possible by key players, including non-government and not-for-profit organizations.

Another aspect that is primarily missing in our results, but is a translational aspect of our results, is the need for rapid and affordable diagnostic assays for RGDs. The current diagnosis gold standard for RGDs is either WGS or WES, but their cost is not bearable for patients in the South Asian region and some regions of the Eastern Mediterranean Region. Thus, based on the shared ancestry and genetic admixture in these regions, we suggest building a regional database of genetic variants. This database could be useful for cheaper and rapid RGD diagnosis in the region. As the region-specific information is much clearer by our discovery of tens of South Asian-enriched pathogenic variants, other previous studies [98,99] have also reported it. Therefore, we suggest building a broader regional catalogue of pathogenic and South Asian-enriched pathogenic variants, and these efforts should be accelerated by financial and scientific incubations. Moreover, subsidies should be announced for organizations, companies, and startups that can take up the challenge of building rapid and affordable diagnostics. These could be genotype arrays, multiplexed PCRs, cheaper and rapid sequencing methods, population-specific or pan-genomic gene panels. In addition, following diagnosis, genetic counselling and efforts to ensure treatment availability are needed. All these things together could be compiled in one RGD policy, which is immediately needed in Punjab and Pakistan and also in the whole region. This policy should include not only a document but also several practical aspects, including human resource training, specifically in clinical genetics, molecular pathology, and genetic counselling. Following this policy, a mass carrier or prenatal screening programme should be launched in families with history of RGDs in extended families or higher incidence of consanguinity. Similarly, clinicians, government and not-for-profit organizations should build a consortium to collaborate with ongoing academic and pharmaceutical research to seek novel drugs and the participation of patients of South Asian origin in ongoing clinical trials.

Although our study is unique in terms of the RGD cohort of Punjabi and other ethnic origins, several limitations are also associated with our study. The largest limitation was the small sample size for each RGD, except for a few, and the lack of data for the adult onset of RGDs. Inclusion of more RGD families in this registry would enhance our understanding of both aspects in the future. In addition, ethnicity- and caste-specific samples are also necessary to understand genetics of RGDs in these groups. Due to small sample sizes and non-random snowball sampling, we could not estimate the population prevalence of these RGDs at this time. Instead, we reported a pathological allele frequency representative of RGD share in the current registry version. We hope we will be able to obtain more randomized data on RGDs and calculate RGD-specific prevalence in future. Moreover, the current registry only includes RGDs of pediatric onset, we will also expand it to RGDs of adult onset in the near future. Our data of abortions includes both spontaneous losses and elective terminations, a future study with clinical differentiation of both among RGD carrier mothers would define the pattern more clearly. For this, we plan to collaborate with other tertiary care hospitals in Punjab. Although we do not have sufficient funds to initiate drug discovery, we would like to collaborate with ongoing clinical trials for RGDs, as we have patients registered to support clinical trials for future medicines.

## 4. Materials and Methods

### 4.1. Study Settings

The study was conducted from January 2021 to December 2023 at the PTGDPRI at Sir Ganga Ram Hospital, Lahore, Pakistan and National Center of Excellence in Molecular Biology (CEMB), University of Punjab (PU), Lahore, Pakistan. An informed consent was obtained from all registered families and the study was approved by institutional review board of CEMB under letter no. IRB-5/24, dated 19 December 2024.

### 4.2. Sampling Technique

The snowball sampling technique was used to include the maximum possible RGDs in the registry presented in this study. The PTGDPRI offers chorionic villus sampling (CVS) for prenatal screening of genetic diseases; thus, RGD carrier families walked in for CVS have been registered in this registry since January 2021 after informed consent.

### 4.3. Inclusion and Exclusion Criteria

The inclusion criterion for our study (also the registry) was any family with a confirmed RGD diagnosis. All diagnoses were based on WGS, WES, or targeted capture sequencing. In case a patient was suspected of having an RGD, the patient was followed up until a final diagnosis was made. Patients with suspected disease for whom a final diagnosis was not made by 31 December 2023, were not included or presented in this study.

### 4.4. Patient Data Collection

Patient data were recorded using a registry questionnaire, including final RGD diagnosis, demographic information such as ethnicity, caste, district, consanguinity, family income, and total number of children in the family, including affected, died and normal children. In addition, self-reported GPA information, was also recorded. In case, when more than one phenotype of an RGD occurs, a common phenotypic umbrella name is used in main figures; however, phenotypic subtypes are discussed in text and Supplementary Data.

### 4.5. Genetic Data

RGD genotypes were registered for 91 families in the current registry. This genotype information was obtained from trio massive parallel NGS (WGS, WES, or targeted-capture sequencing) of the proband and both parents. WGS was performed either by referring clinician’s office participating in a genetic testing programme or a third party commercial clinical service provider, and only genotypes of potential clinical interest, including pathogenic, likely pathogenic, or variants of unknown significance, were registered.

### 4.6. Genetic Methods

For all patients, probands, or parents, a blood sample, buccal swab, saliva, or placental tissue was collected and sent to a CAP and/or ISO15189-certified commercial genetic testing laboratory. These laboratories applied commercial testing and in-house pipelines to identify the variants. A summary of these steps is provided below. Post- extraction, genomic DNA was enzymatically fragmented, and target regions were enriched using DNA capture probes. These probes captured either whole genome, whole exome or a set of targeted genes. In all cases of WES, Twist Human Core Exome Plus kit (Twist Bioscience, San Francisco, CA, USA) was used to capture approximately 41 Mb of the human coding exome (targeting ≥ 98% of the coding RefSeq from the human genome build GRCh37/hg19) as well as mitochondrial DNA. In all patients except one, the Illumina short-read sequencing platform was used to sequence the whole genome, exome, or targeted genes at 20X or higher coverage for the nuclear genome and up to 1000X for the mitochondrial genome. Only coding genes and their exon-intron boundaries were targeted in case of WES and targeted-capture sequencing. All variants with a minor allele frequency (MAF) of less than 1% in the gnomAD database and disease variants reported in HGMD^®^ and ClinVar were considered.

Commercial laboratories used in-house bioinformatics pipelines for read alignment to the GRCh37 or GRCh38 human genome assembly and revised Cambridge Reference Sequence (rCRS) of the Human Mitochondrial DNA (mtDNA). Read alignment followed variant calling using DRAGEN (v4.2.4 Illumina, San Diego, CA, USA) or a similar in-house tool to identify short or structural variants. These variants were annotated using ClinVar, dbSNP, gnomAD, or OMIM^®^. Structural variants were annotated using in-house pipelines. Once variants were annotated with appropriate identification and allele frequencies, variants with minor allele frequency (MAF) less than 1% were retained for downstream analysis. The copy number variation analysis was based on the coverage of depths. All potential patterns of inheritance were considered during variant discovery. In addition, the provided family history and clinical information were used to evaluate the identified variants with respect to their pathogenicity. Identified variants were reported as pathogenic, likely pathogenic, VUS, likely benign, or benign under the joint standards and guidelines of the ACMG/AMP [17], SVI recommendations for ACMG/AMP guidelines from The ClinGen Sequence Variant Interpretation Group, and/or ACMG secondary findings (SF) version 3.0 [60]. Only variants related to the clinical phenotype of the patient were reported.

### 4.7. Healthy Controls Data

Consanguinity data of randomly sampled 1011 healthy couples without RGDs were obtained from the Punjab Consanguinity Survey [61]. These data were used to compare consanguinity between RGD carrier families and healthy couples.

### 4.8. Consanguinity Analysis

We conducted a descriptive comparison of consanguinity patterns in RGDs. Data were collected from demographic sources based on their link to consanguineous marriages. We report the frequencies of the overall rates of cousin marriages. This analysis provided a more precise breakdown of the proportion of marriages between first cousins, second cousins, relatives, and unrelated individuals in RGD carrier families. The consanguinity information of RGD carrier families and healthy couples from the Punjab Consanguinity Survey [61] was tested for the association of consanguinity with RGDs using association and effect size tests. The Punjab Consanguinity Survey consists of 1011 families randomly sampled from nine districts in Southern, Northern, and Central Punjab, representative of region-wide consanguinity rates.

### 4.9. Variant Annotation

After recording, each patient/proband and RGD carrier family was classified in the registry, and primary, secondary, and VUS information was recorded in the registry. All variants were annotated against their registered single nucleotide polymorphism (SNP) identification numbers (rsIDs) allocated by dbSNP [100] or nucleotide identification (NM) allocated by the ClinVar database. The allele frequency of each registered variant in our registry was calculated using the following formula:

$$Allele\;Frequency\;\left(\%\right)=\;\frac{\sum_{i=1}^{n}\eta_{i}\;}{2N}\times 100$$

where $\sum_{i=1}^{n}\eta_{i}$ is the sum of alleles of carriers for each variant and 2*N* is the sum of ploidy (total chromosomes) in the registry.

The allele frequency of each registered variant worldwide was annotated using rsIDs from the gnomAD v4.0 database to understand the global distribution of variants and identify population-specific variants.

### 4.10. Statistical Analysis

We performed descriptive statistics, including frequencies and percentages, for qualitative variables. Central tendency and distributions were measured using the mean and standard deviation. Crosstabs were constructed between the variables to establish relationships. Pearson’s chi-square test was applied to determine the association between qualitative variables with a *p*-value threshold of 0.05. For effect size measurement, we used OR, the reciprocal of OR, RR, and the reciprocal of RR. SPSS v22.0, Microsoft Excel 2016, GraphPad Prism 10, or R Studio (2025.05.1+513) packages *tidyr* and *ggplot2* were used for these analyses and visualizations.

## 5. Conclusions

This registry provides the first foundational dataset for RGDs in the Punjabi population and represents a model for integrating molecular epidemiology with public health genetics that could guide provincial and national policy. The Punjabi population of Pakistan illustrates how genetic, socioeconomic, and cultural factors converge to amplify the burden of RGDs in this population. High consanguinity, limited diagnostic access, and the absence of policy infrastructure act synergistically to sustain this burden in Pakistan. There is an urgent need to develop cost-effective diagnostic assays that target RGD-associated variants prevalent in South Asian and Middle Eastern populations to minimize inequity in access to healthcare. Translating advanced molecular technologies into affordable diagnostic platforms is crucial for achieving this goal. Expansion of this registry into a national genomic surveillance programme could enable early detection, equitable data sharing, and translational research collaborations. Comparative allele frequency analyses using the gnomAD database further demonstrated that several pathogenic variants are exclusive to South Asian ancestry, underscoring regional founder events and the critical need to include underrepresented populations in global RGD genomics research.

## Figures and Tables

**Table 1:** The number of diagnoses by different genomic techniques.

Number of Diagnoses Made by Short and Structural Variant Discovery Methods		
	Short Variant Discovery	Structural/Copy Number Variation
Short Variant Discovery	91	35
Copy Number Variation	35	35
**Sequencing Techniques Utilized for Diagnosis**		
Whole Genome Sequencing	Whole Exome Sequencing	Targeted Capture Sequencing
8	56	27

**Table 2:** Classification of variants identified in patients in current study.

Variant Class	Primary Variants	Secondary Variants	Variants Of Unknown Significance
Frameshift Likely Pathogenic	1	5	0
Frameshift Pathogenic	6	0	0
In Frame Pathogenic	1	0	0
In Frame Uncertain Significance	2	1	3
Large Inversion	1	0	0
Likely Pathogenic	5	11	0
Loss Like Pathogenic	2	0	0
Loss Pathogenic	1	0	0
Missense Pathogenic	18	18	0
Missense Likely Pathogenic	14	6	0
Missense Uncertain Significance	13	7	20
Nonsense Pathogenic	12	1	0
Nonsense Likely Pathogenic	5	2	0
Pathogenic	1	1	0
Silent Pathogenic	0	1	0
Silent Uncertain Significance	1	0	1
Splicing Likely Pathogenic	1	0	0
Splicing Pathogenic	3	2	0
Splicing Uncertain Significance	1	0	1
Vus	3	3	6
Total	91	58	31

## Data Availability

The original contributions presented in this study are included in the article/Supplementary Material. Further inquiries can be directed to the corresponding author.

## Supplementary Materials

The following supporting information can be downloaded at https://www.mdpi.com/article/10.3390/ijms27010206/s1.
