# Supplementary material for: Uncovering the Molecular Signatures of Rare Genetic Diseases in the Punjabi Population
Source: Int J Mol Sci. 2025 Dec 24;27(1):206. doi: 10.3390/ijms27010206 (PMC12785397; doi:10.3390/ijms27010206)
Supplement: Supplementary file 1 [file ijms-27-00206-s001.zip › List of supplementary tables.pdf]

List of supplementary tables

Table S1: List of rare genetic diseases in study cohort

Table S2: Allele frequencies of variants in Pakistani Populatio
