# Supplementary material for: Uncovering the Molecular Signatures of Rare Genetic Diseases in the Punjabi Population
Source: Int J Mol Sci. 2025 Dec 24;27(1):206. doi: 10.3390/ijms27010206 (PMC12785397; doi:10.3390/ijms27010206)
Supplement: Supplementary file 1 [file ijms-27-00206-s001.zip › Supplementary Figures.pdf]

List of Supplementary Figures:

Supplementary Figure S1: Geographical distribution of RGDs

A: The mapping of RGD families across the Punjab Province illustrates the spatial distribution of families affected by RGDs, highlighting regional clustering and population-specific concentration patterns

B: The geographical distribution of RGDs in various districts showing areas with higher and lower disease occurrence

Supplementary Figure S2: Classification of rare and ultra-rare diseases

This figure illustrates classification of rare & ultra-rare diseases according to their occurrence rates, distinguishing rare diseases from ultra-rare diseases to better understand their epidemiological and clinical distribution

Supplementary Figure S3: Consanguinity comparison among Ethnic Groups

This pattern shows a comparison of consanguinity and ethnicity among families affected by RGDs showing variation in consanguineous marriage rates across ethnic groups

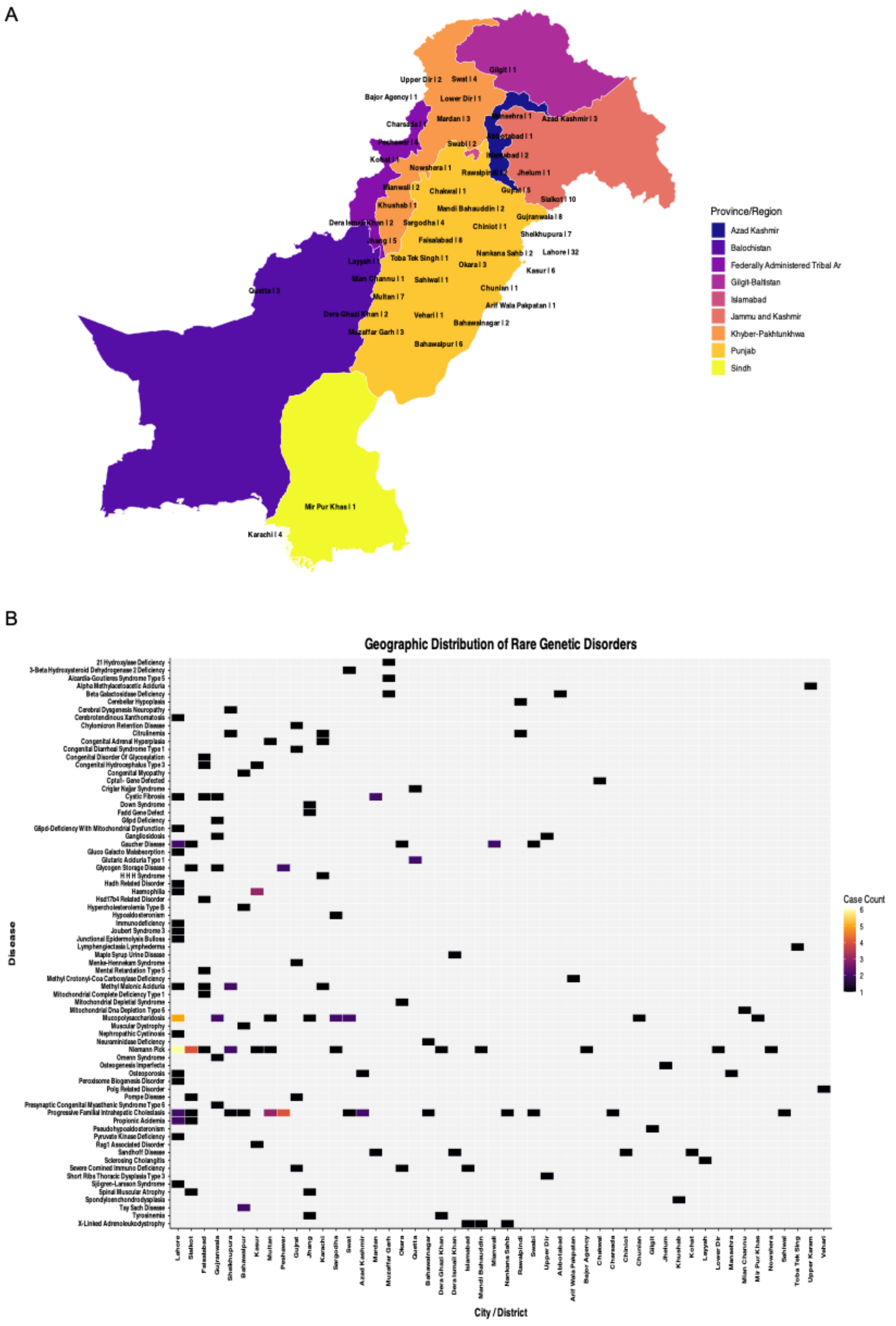

Supplementary Figure S1

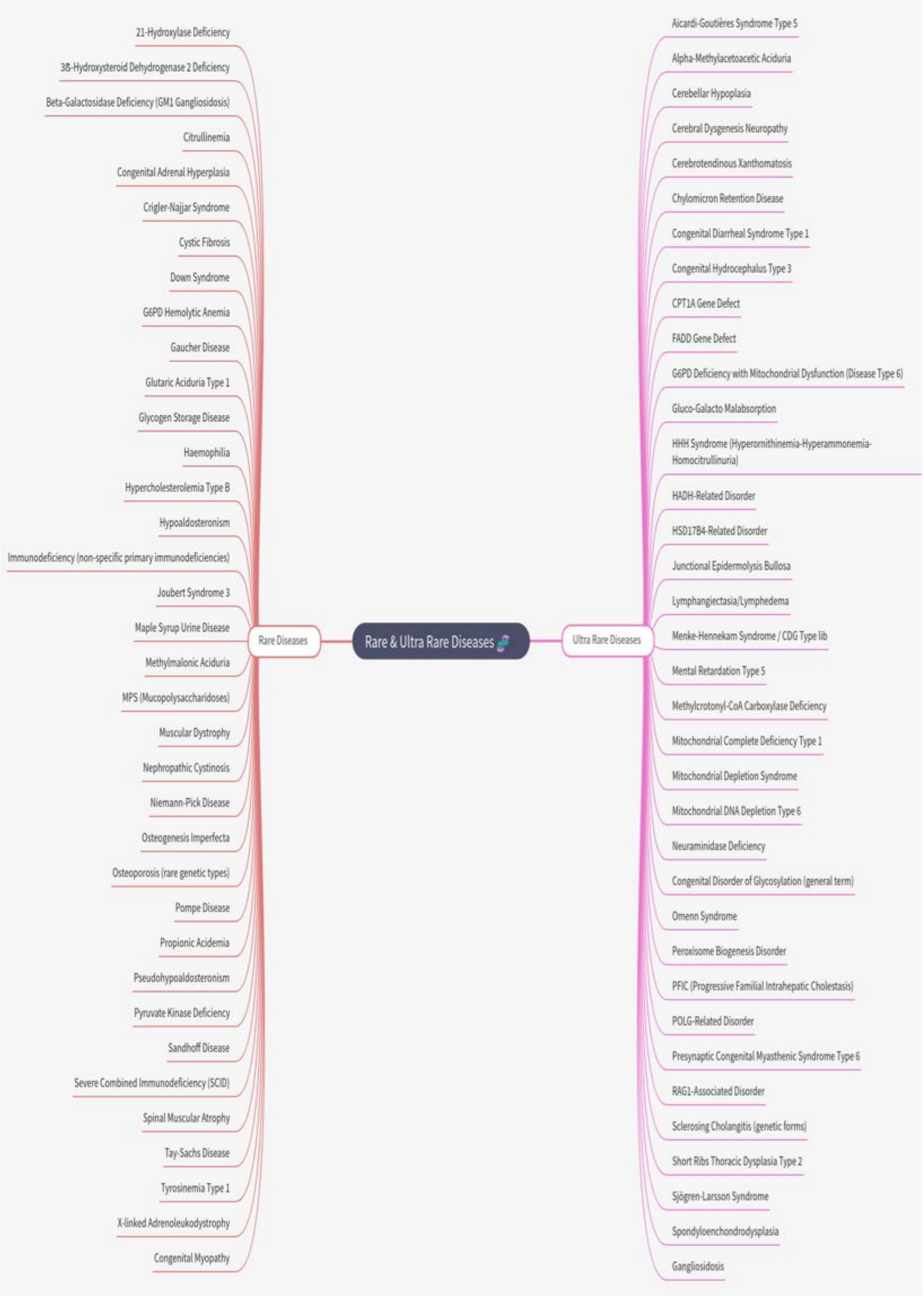

Supplementary Figure S2

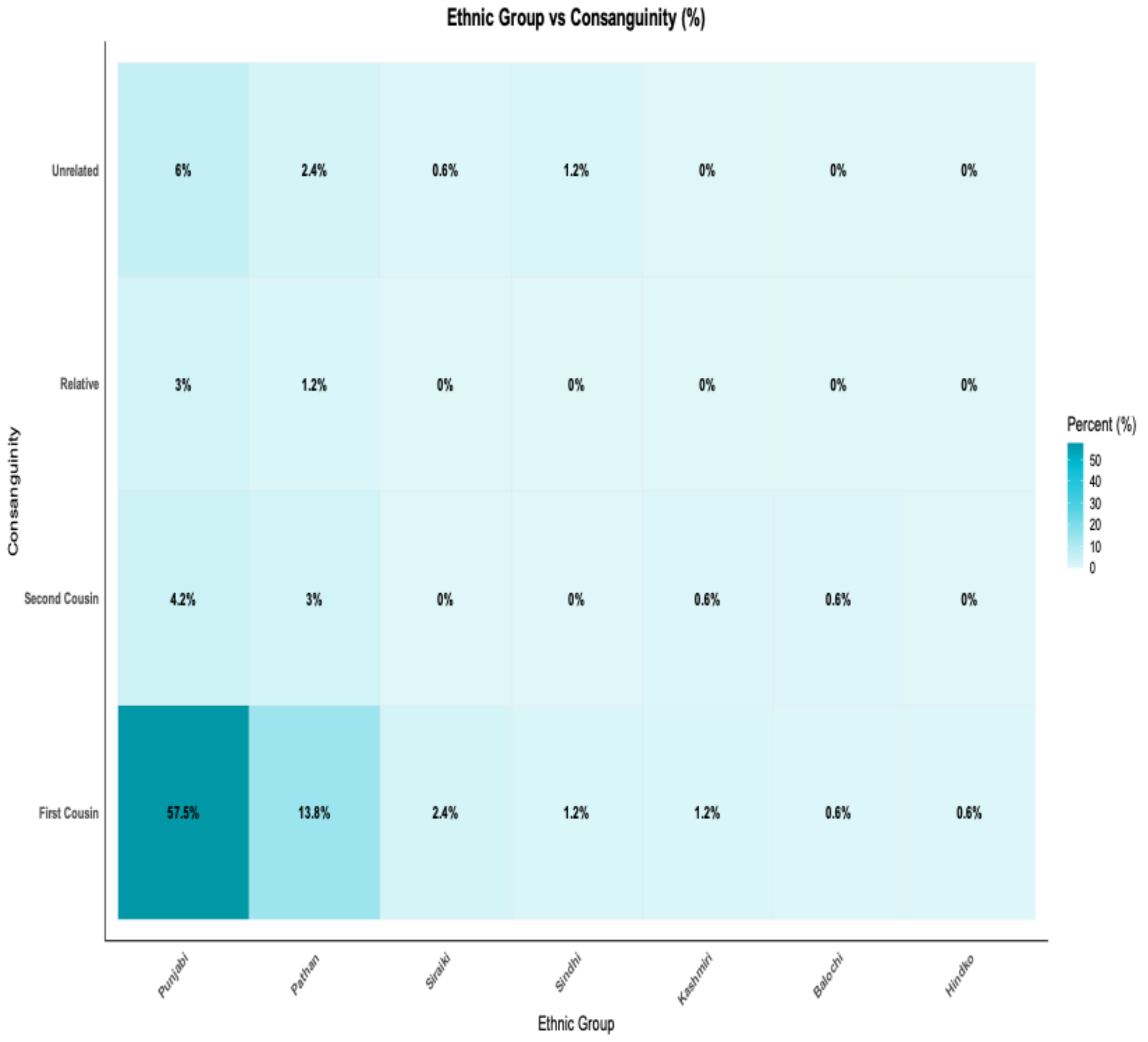

Supplementary Figure S3
